# Dissecting the causal association of diet with thyroid cancer: a systematic review with meta-analysis and mendelian randomization analysis.

**DOI:** 10.3389/fnut.2025.1664129

**Published:** 2025-09-17

**Authors:** Chao Kang, Yongyao Du, Jiaxin Li, Yi Yang, Jingping Li, Manping Zhou, Jiaojiao Shi, Ning Lin, Xin Ma, Xiaoli Peng

**Affiliations:** ^1^School of Public Health, Chengdu Medical College, Chengdu, Sichuan, China; ^2^Department of Clinical Nutrition, The General Hospital of Western Theater Command, Chengdu, Sichuan, China; ^3^School of Public Health, Medical College of Soochow University, Suzhou, Jiangsu, China; ^4^Department of Rehabilitation Medicine, The General Hospital of Western Theater Command, Chengdu, Sichuan, China; ^5^Sichuan Provincial Key Laboratory of Philosophy and Social Sciences for Intelligent Medical Care and Elderly Health Management, Chengdu Medical College, Chengdu, Sichuan, China

**Keywords:** food groups, dietary pattern, thyroid cancer, meta-analysis, mendelian randomization analysis

## Abstract

**Background:**

Diet is a recognized risk factor for cancer. Recently, the role of improving thyroid-related functions through diet has been questioned. This systematic review investigates the relationship between food groups/dietary patterns and thyroid cancer.

**Methods:**

We conducted a systematic search of the literature through April 2025 in the PubMed, Scopus, Web of Science, and Embase database following PRISMA guidelines. ORs, HRs or RRs with 95% CIs were extracted as effect sizes and publication bias was assessed using funnel plots. Additionally, we conducted mendelian randomization (MR) analysis by selecting dietary factors (including nutrients) associated with thyroid cancer as exposure data to complement the results of meta-analysis.

**Results:**

We collected data from 16 cohort and 21 case-control studies that met the collection criteria. Meta-analysis found that high consumption of fish and alcohol-containing beverages was associated with a reduced risk of thyroid cancer, whereas consumption of high amounts of refined cereal and nitrates increased thyroid cancer risk (*P* < 0.05). Our MR analysis data showed that some specific food items, especially seafood (like oily fish) might be the protective factors for thyroid cancer, which strengthen the previous meta-analysis results.

**Conclusion:**

This comprehensive study investigated the relationships between dietary factors and thyroid cancer risk, synthesizing findings from a meta-analysis of observational studies and MR analysis to estimate causal associations. Consistently, both the meta-analysis and MR analysis revealed that consumption of certain types of fish may be linked to a decreased risk of thyroid cancer.

**Systematic review registration:**

https://www.crd.york.ac.uk/PROSPERO/, identifier CRD420251101506.

## 1 Introduction

The incidence of thyroid cancer has continued to rise over the past few decades, and the burden of thyroid cancer is concentrated in women (1). According to the Global Cancer Incidence Statistics 2020, thyroid cancer is the ninth most prevalent cancer and is three times more prevalent in women than in men (2). A collaborative study analyzing data from 13 cancer registries in China, Japan, and Korea found that the age-specific incidence curves for Chinese and Korean individuals were characterized by an inverted U-shape (3). Dietary factors are considered putative risk factors for the development of cancer at different sites, while nutritional factors play an important role in the pathogenesis of different metabolic diseases. In recent years, many studies have attempted to establish a relationship between diet and thyroid cancer, but remain inconclusive. Dietary pattern appears to alter the risk of thyroid cancer. A diet low in starchy foods, products rich in salt, fat and sugar, and high in cruciferous/non-cruciferous vegetables, milk, dairy products and seafood can prevent thyroid cancer (4). One study showed a significant negative correlation between a dietary pattern rich in fruits and vegetables and thyroid cancer (5). However, another study revealed no significant association between vegetable/fruit intake and different types of thyroid cancer (6). Similarly, other studies did not present strong evidence for the association between dietary intake of selenium or other micronutrients and thyroid cancer risk (7). As for daily beverages such as alcohol, coffee and tea, one study demonstrated that the risk reduction associated with alcohol was eliminated after adjusting for smoking factors, and that thyroid cancer risk was not associated with coffee or tea consumption (8), but a large cohort demonstrated a potentially protective effect of alcohol consumption against thyroid cancer (9).

There has been a growing interest in the impact of dietary pattern or food intakes on thyroid cancer. However, in each clinical trial, various food items and different dietary patterns caused mixed effects. Meta-analysis needs to be updated for an overview of the current evidence on the association between diet factors (food groups and dietary patterns) and thyroid cancer. Besides, mendelian randomization (MR) uses genetic variants as instrumental variables (IVs) to estimate the causal effect of an exposure (diet) on an outcome (thyroid cancer) (10). The main objective of this meta-analysis is to outline the relationship between food groups [vegetables, fruits, meat, dairy products, tea, alcohol, seafood (fish, saltwater fish, shellfish and fresh-water fish)], dietary patterns and thyroid cancer, then MR was used to complement the observational findings. To our knowledge, this is among the first studies to synergistically combine meta-analysis and two-sample MR analysis, leveraging the complementary strengths of observational evidence (meta-analysis) and causal inference (MR). While meta-analysis provides a comprehensive overview of dietary associations, MR mitigates confounding and reverse causation, offering a robust framework to infer causality.

## 2 Materials and methods

### 2.1 Search strategy

This systematic review used the principles stated in the Preferred Reporting Items for Systematic Reviews and Meta-Analysis (PRISMA; Supplementary File 1) (11). The meta-analysis has been registered on PROSPERO (CRD420251101506). This screening process was independently completed by two reviewers (YD and JL). A systematic search was performed in MEDLINE (via PubMed), Scopus (via Elsevier), Web of Science-Science Citation Index and Social Sciences Citation Index (via Clarivate), and Embase (via Elsevier) on April 2025, with no restrictions regarding language or year of publication. The search included the following keywords: “’food groups” (vegetables, fruits, meat, dairy products, alcohol, starchy, fish, saltwater fish, fresh-water, fish, shellfish, nitrate, tea, seafood, seaweed) OR “dietary pattern” (healthy dietary pattern, unhealthy dietary pattern) AND “thyroid cancer” AND “incidence” OR “risk.” We also conducted manual searches of preprint platforms (medRxiv and Research Square) and other databases (CINAHL, China National Knowledge Internet Database, Wanfang database). We manually searched the gray literature through ClinicalTrials.gov, Cochrane Central Registry of Controlled Trials, and Web of Science: Conference Proceedings Citation Index-Science (via Wiley) to identify completed but unpublished studies that met our eligibility criteria for reducing publication bias. The authors were contacted to obtain missing data.

### 2.2 Eligibility criteria

We included the studies that met the criteria: (1) Article assessing the relationship between food groups or dietary patterns and thyroid cancer. (2) Thyroid cancer incidence as the outcome. (3) Case-control, cohort studies or randomized controlled trials. The following exclusion criteria were applied: (1) Studies on cell-level, animal or model studies. (2) Letters, case reports, conference reports, laboratory studies, editorials and any review.

### 2.3 Data extraction

A standardized data collection sheet was designed and created in Microsoft Excel. Two reviewers (YD and JL) performed the data extraction for all included articles. The detailed information collected from each included study was as follows: first author, year of publication, country, study design, sample sizes of cases/controls, duration of follow-up (this is appropriate for cohort studies), exposure intake of study/control groups, and potential adjustment factors (Table 1). In cases of missing information, the corresponding author was contacted via email to request the missing data.

**TABLE 1:** Characteristics of the studies included in this review.

No.	Author, Year	Country	Study design	Case/subject Case/control Follow-up	Age	Gender (male/ female)	Group comparisons	Main outcome	No. of cancer type	NOS
#01	Fioretti et al. (49)	Italy	Case-control	399/617	16–72/16–74	Cases: 108/291; controls: 190/427	Refined cereal: tertile (the highest vs. the lowest); β-carotene: tertile (the highest vs. the lowest).	60% of thyroid cancer cases may be caused by some combination of identifiable factors or risk related factors, such as any history of benign thyroid disease, a history of radiation therapy, long-term residence in an area where goiter is prevalent, and some basic dietary indicators.	Papillary: 274; follicular: 69	8
#02	Galanti et al. (31)	Sweden	Case-control	246/440	18–75	Cases: 59/187; controls: 107/333	Milk (glass), yogurt/acidified milk (dl), butter (tspn), all grains, all starchy foods, bread (slices), all fish, salt-water fish and shellfish, fresh-water fish, fish products, roe, caviar, all meat, all vegetables, vegetables (excluding cruciferous, cruciferous), all fruit (piece), apple, citrus fruit, coffee: tertiles alcohol: the highest tertile of alcohol intake compared with the lowest tertile (data not shown).	1. High consumption of butter and cheese was associated with increased risks. 2. High consumption of cruciferous vegetables was associated with increased risk only in persons who ever lived in such areas. 3. A decreased risk was associated with consumption of iodized salt in northern Norway, and with use of iodized salt during adolescence among women.	Papillary: 209; follicular: 37	7
#03	Kilfoy et al. (50)	America	Prospective cohort	370/490194, 7 years	50–71	Cases: 170/200	Nitrate and nitrite intake: highest quintile versus lowest quintile	1. Among men, increasing nitrate intake was positively associated with thyroid cancer risk, however, we observed no trend with intake among women. 2. Nitrite intake was not associated with risk of thyroid cancer for either men or women.	Papillary: 258; follicular: 64; medullary: 7	8
#04	Mack et al. (22)	America	Case-control	292/292	15–54	All female	Shellfish, saltwater fish, freshwater fish, turnips or rutabagas: the frequency of consumption (less than few times per year; few times per year; few times per month; few times per week or more); taking multivitamins: the frequency of consumption (never used regularly; >0–2 years; >2–10 years; >10 years); caffeinated coffee: the frequency of consumption (none; 1 cup per day; 2–3 cups per day; 4–5 cups per day; 6 or more cups per day); caffeinated tea, caffeinated sodas, beers, glasses of wine, shots of whiskey: the frequency of consumption (none; 1–2 cups per day; 3 or more cups per day): the frequency of consumption (none; 1–2 cups per day; 3 or more cups per day)	1. Thyroid cancer risk was not associated with fish consumption, and consumption of certain vegetables, wine and tea reduced the risk of cancer. 2. Milk, beer and spirits, and coffee were not associated with thyroid cancer risk.	Papillary: 240; follicular: 16	6
#05	Michikawa et al. (51)	Japan	Prospective cohort	134/52545, 14.5 years	40–59	All female	Seaweed consumption: food-frequency questionnaire and divided into three categories: 2 days/week or less (reference); 3–4 days/week; and almost daily	This study identified a positive association between seaweed consumption and the risk of thyroid cancer (especially for papillary carcinoma) in postmenopausal women	Papillary: 113	8
#06	Nguyen et al. (52)	Korea	Prospective cohort	138/13835, 7.6 years	50.9 ± 7.7/ 53.3 ± 8.6	Cases: 18/120; controls: 4904/8931	Meal frequency: 3 meals/day for ≥5 days/week (yes or no); meal duration: ≥10 min (yes or no); meat and eggs: ≥5 times/week (no meat or amount of meat ≥ the size of 2 ping-pong balls and ≥1 egg or amount of meat < the size of 2 ping-pong balls and <1 egg); seafood ≥ 3 times/week (yes or no); tofu or soy milk ≥ 3 times/week (yes or no); vegetables, seaweed, mushrooms (except kimchi) every meal (yes or no); fruits ≥ 5 days/week (yes or no); milk or dairy products ≥ 5 days/week (yes or no); taste salty when eating out (yes or no); tend to eat salty food (yes or neutral or no); grilled meat frequency (never, sometimes, often, N/A (not applicable)	Consuming milk and/or dairy products 5 or more days a week and having a meal duration longer than 10 min could be protective factors against TC, especially in individuals aged ≥50 years, women and non-smokers.	Not mentioned	7
#07	O’Grady et al. (7)	America	Prospective cohort	592/565806, 4,406,634 person-years	50–71	N: 287944/194683; cases: 257/335	Selenium, vitamin C, betacarotene, calcium, folate, vitamin E, vitamin D, magnesium, selenium, vitamin C, betacarotene, calcium, folate, vitamin E, vitamin D, magnesium, zinc: quintiles	No association between dietary intake of selenium or other micronutrients and thyroid cancer risk	Not mentioned	8
#08	Takezaki et al. (53)	Japan	Case-control	94/22666	20–79	All female	Western breakfast, raw vegetable, fruit, green vegetable, cabbage, lettuce, carrot, tofu, miso soup, milk, egg, chicken, beef, pork, fish: intake frequency (low, medium, high)	1. Frequent coffee consumption may be involved in a decreased risk of thyroid disease. 2. Other dietary factors did not show significant increased or decreased risk.	Papillary:91; follicular: 3	7
#09	Xhaard et al. (54)	France	Case-control	747/815	<15	Cases: 172/633; controls: 197/679	Fresh dairy products or fresh milk: assessed in “never”/“ever” categories and by quartiles of quantities; leafy vegetable: assessed in “never”/“ever” categories and by quartile of quantities	1. The DTC risk was slightly higher for participants who had consumed locally produced leafy vegetables. However, this association was not stronger in the more contaminated areas than in the others. 2. Conversely, the reported consumption of fresh dairy products was not statistically associated with DTC risk.	Not mentioned	8
#10	Xiao et al. (55)	America	Prospective cohort	586/491254, 9 years	50–71	N: 234597/198593; cases: 252/334	Dietary intakes of total flavonoids, flavonoid subtypes, and flavonoid-rich foods: quintiles.; tea: frequency of consumption (0; ≤1 cup/week; >1 cup/week –1 cup/day; >1–3 cup/day; >3 cup/day); oranges and tangelos, orange and grapefruit juice, legumes, grape, banana: median intake	1. Thyroid cancer risk was inversely associated with dietary flavan-3-ols, but positively associated with flavanones. 2. Other classes of flavonoids and total flavonoids; were not associated with thyroid cancer risk.	Not mentioned	7
#11	Zamora-Ros et al. (56)	Spain	Prospective cohort	748/476108, 14 years	35–70	Cases: 82/666	Fish: quartiles (highest quartile vs. lowest quartile); shellfish: 3 groups (non-consumers and those below and above the median of consumers)	The intake of fish and shellfish was not associated with differentiated TC risk	Papillary: 601; follicular: 109	8
#12	Zamora-Ros et al. (56)	Spain	Prospective cohort	748/476108, 14 years	35–70	Cases: 82/666	Total F&V, vegetables, fruits, fruit juices: quartiles (highest quartile vs. lowest quartile)	1. . No significant association between F&V intakes and differentiated TC risk. 2. . A positive trend with fruit juice intake was observed in TC.	Papillary: 601; follicular: 109	8
#13	Zamora-Ros et al. (34)	Spain	Prospective cohort	748/476108, 14 years	35–70	Cases: 82/666	Coffee: cohort-wide quartiles. Tea: cohort-wide tertiles	Coffee and tea consumption were not associated with TC risk.	Papillary: 601; follicular: 109	8
#14	Zamora-Ros et al. (57)	Spain	Prospective cohort	712/521324, 14 years	35–70	Cases: 74/638	Tea, coffee, alcoholic beverages (beer and wine), sweetened beverages, and milk and dairy products: quartiles	Consumption of sweetened beverages was associated with a higher risk of differentiated thyroid cancer.	Papillary: 573; follicular: 108	8
#15	Jung et al. (23)	Korea	Case-control	226/226	20–70	All female	Total vegetables, raw vegetables, total fruits and all single fruits: quartiles of daily intake (g/d) and average consumption frequency (times/week)	High consumption of raw vegetables, persimmons and tangerines may decrease thyroid cancer risk and help prevent early-stage thyroid cancer.	90% papillary	7
#16	Braganza et al. (58)	America	Prospective cohort	325/292152, 10 years	12–13/41–62	Cases: 143/182; controls: 170832/121320	Grains, vegetables, fruit, dairy, chicken or turkey, red meat, canned tuna, butter and/or margarine and sweet baked goods, broccoli: the highest to the lowest quartiles	1. Adolescent intakes of chicken/turkey and sweet baked goods were positively associated with thyroid cancer risk, while intake of butter/margarine was inversely associated with risk. 2. Mid-life intake of sweet baked goods was non-significantly associated with an increased risk of thyroid cancer but intake of butter/margarine was inversely associated with risk. 3. Among men, higher adolescent consumption of canned tuna was positively associated with risk of thyroid cancer, and greater mid-life intake of broccoli was associated with a twofold increased risk. 4. Iodine-rich foods may influence thyroid cancer risk.	Papillary: 240; follicular: 60	8
#17	Sangsefidi et al. (46)	Iran	Case-control	41/268	38.31 ± 12.52/ 41.82 ± 13.15	Cases: 5/36; controls: 40/228	Dietary pattern scores: tertiles	1. The western dietary pattern significantly increases the odd of DTC. 2. The traditional, transitional, healthy dietary patterns did not have any association with DTC.	Papillary: 33; follicular: 6; medullary: 2	7
#18	Aschebrook-Kilfoy et al. (59)	America	Prospective cohort	164/73153, 9 years	40–70	All female	Nitrate and nitrite: the highest quartile vs. the lowest quartile	1. No association between dietary nitrate intake and thyroid cancer as hypothesized. 2. An approximately 2-fold increased risk of thyroid cancer associated with the highest quartile of nitrite intake.	Not mentioned	9
#19	Cléro et al. (60)	France	Case-control	229/371	<56	Cases: 26/203; controls: 47/324	Dietary pattern (traditional Polynesian and Western): 3 categories (the highest tertile vs. the lowest tertile); goitrogenic food: tertiles (the highest tertile vs. the lowest tertile)	1. No association between a western dietary pattern and the risk of thyroid cancer in French Polynesia. 2. A traditional Polynesian dietary pattern led to a weak reduced risk of thyroid cancer.	Papillary: 177; follicular: 52	8
#20	Cléro et al. (61)	France	Case-control	229/371	<56	Cases: 26/203; controls: 47/324	Fish consumption (g/day), shellfish consumption (g/day), total consumption of food from the sea (g/day): tertiles dietary iodine: tertiles	A higher dietary iodine intake is significantly associated with a decreased risk of thyroid cancer	Not mentioned	8
#21	Fiore et al. (4)	Italy	Case-control	106/217	47 ± 14/42 ± 17	Cases: 23/83; controls: 65/152	Starchy foods; sweets cruciferous vegetables; non-cruciferous vegetables; meat and meat products; milk and dairy products; fish and seafood: 4-level scale (never, one time a week, 2–3 times a week, every day of the week).; Products rich in salt and fat, legumes, fruit: using cut off 2 (≤2 = never + 1 time a week and >2 = 2–3 times a week + every day); type (tap water, bottled water) and quantity of drinking water:1/2 L, 1 L, 2 or more liters; food supplements, iodized salt and vitamin: use (yes/no); caffeinated drinks: 3-level scale (never, 1–3 cups/die, 4– ≥6 cups/dies)	A diet with a limited consumption of starchy foods, products rich in salt, fat and sugar and a higher consumption of cruciferous/non-cruciferous vegetables, milk and dairy products and seafood could be protective toward thyroid cancer.	Not mentioned	7
#22	Hallquist et al. (62)	Sweden	Case-control	180/360	20–70	Cases: 48/123	Cabbage, green vegetables, root vegetables, meat, fish, shellfish: frequency (I: some times a week; II: several times a week)	1. Eating cabbage or green vegetables did not affect thyroid cancer risk. 2. Intake of root vegetables after age 20 was associated with a significant increase in risk.	Papillary: 107; follicular: 27; mixed (papillary/ follicular): 20; medullary: 7	8
#23	Haslam et al. (63)	America	Longitudinal cohort study	27/108, 16.4 ± 3.1 years	Mean: 32.0/31.8	Cases: 9/18; controls: 36/72	PCB exposure: high (>7.0 mg), medium (1.01–7.0 mg), and low (≤1 mg); omega-3 fatty acid intake from Great Lakes fish: the highest quartile vs. the lowest quartile	No significant associations between fish consumption, short-term estimated omega-3 fatty acids, or estimated PCB consumption from Great Lakes fish and the development of thyroid cancer	Not mentioned	7
#24	Hoang et al. (21)	Korea	Case-control	117/173	52.5 ± 8.0/ 51.7 ± 8.1	Cases: 6/111; controls: 33/140	Dietary seaweed and iodine intakes: quartile groups of high (greater than median amount) and low (less than the median amount) consumption	The protective effects of dietary gim and iodine intake against thyroid cancer risk.	Not mentioned	6
#25	Horn-Ross et al. (64)	America	Case-control	608/558	20–74		Fish consumption: the highest vs. lowest quintile	Multivitamin pills were associated with a reduced risk of papillary thyroid cancer.	Papillary: 544; follicular: 28; medullary: 5	9
#26	Kim et al. (65)	America	Case-control	462/498	21–84	Cases: 87/375; controls: 1154/344	Fiber, multivitamins, beta-carotene, vitamins A/C/E, and calcium supplements: the frequency (once a month, once a week, several times per week, daily) and duration (number of years) of use were collected. fruits (apples, pears, bananas, plums, melons, etc.) and vegetables (leafy greens, cabbage, carrot, broccoli, tomatoes, etc.): the quantity (number eaten) and frequency (number per day, week, or year)	1. No significant associations were observed between dietary vitamin supplementation and overall thyroid cancer risk. 2. The associations between calcium supplements and risk of papillary thyroid cancer were different by tumor size.	Not mentioned	8
#27	Meinhold et al. (9)	America	Prospective cohort	370/489789, 7.5 years	50–71	Cases: 170/200; controls: 291931/197858	Alcohol (drinks): None; <1 per week; 1 – 6 per week; 1 – 2 per day; ≥2 per day	A potential protective role for alcohol consumption in thyroid cancer	Papillary: 258; follicular: 64	7
#28	Memon et al. (66)	Kuwait	Case-control	313/313	15–44	Cases: 75/238; controls: 75/2387	Fish, shellfish, fish products, chicken, mutton and lamb, beef, cabbage, cauliflower, brussels, sprouts, broccoli, green vegetables, pickled, vegetables, fruits: low (never or occasional – few times a year), moderate (1 – 3 times per month or 1 day per week), and high consumption (2 – 4 days per week or 5 – 7 days per week)	1. High consumption of processed fish products, fresh fish and chicken were independently associated with thyroid cancer with significant dose-response relationships. 2. No clear association emerged with consumption of cruciferous vegetables.	Papillary: 186; follicular: 27; medullary: 3	7
#29	Myung et al. (67)	Korea	Case-control	802/802	30–70	Cases: 229/573; controls: 432/370	Alcohol consumption (non-drinker; former drinker; current drinker; ever-drinker)	Alcohol consumption was associated with a decreased risk of TC.	Not mentioned	9
#30	Preston-Martin et al. (68)	America	Case-control	207/207	18–54	All female	Saltwater fish, shellfish, freshwater fish: (never or infrequently; few times a month; few times a week; daily)	TC ate more fish and shellfish, but there was no trend with level of consumption.	Papillary: 173; follicular: 29; medullary: 4	7
#31	Wie et al. (69)	Korea	Prospective cohort	136/7637, 7 years	49.3 ± 8.0/ 48.4 ± 9.2	Cases: 64/72; controls: 4180/3457	Red meat and Na, vegetables and fruits: high intakes vs. low intakes; dietary risk factors: ranged from 0 to 4 points	Red meat consumption and Na (salt) intake were positively associated with thyroid cancer.		8
#32	Truong et al. (70)	France	Case-control	293/345	>18	All female	Salt water fish, brackish water fish, seafood, dairy products, cruciferous vegetables, and starchy foods: quartile (the highest quartile vs. the lowest quartile)	1. Consumption of fish and seafood is not related to thyroid cancer. 2. A positive association between the consumption of cruciferous vegetables and thyroid cancer.	Papillary: 255; follicular: 38	7
#33	Wang et al. (71)	Japan	Prospective cohort	94/35593, 13.2 years	40–79	All female	Seaweed intake frequency: 1–2 times/week or less, 3–4 times/week, and almost daily.	No association between seaweed intake and thyroid cancer incidence in premenopausal or in postmenopausal women.	Papillary: 64	8
#34	Ward et al. (32)	America	Prospective cohort	45/21932, 19 years	55–69	All female	Dietary nitrate: (highest vs. lowest quartile)	1. . An increased risk of thyroid cancer with higher average nitrate levels in public water supplies and with longer; consumption of water exceeding 5 mg/L nitrate-N. 2. . Increasing intake of dietary nitrate was associated with an increased risk of thyroid cancer and with the prevalence of hypothyroidism, but not hyperthyroidism.	80% papillary; 18% follicular	9
#35	Wingren et al. (72)	Sweden	Case-control	104/387	20–60	Cases: 11/93; controls: 187/200	Intake of fish, intake of cruciferous vegetables: Several times a week; once a week; once a month; seldom/never; intake of shellfish: once a week or more; once a month; seldom/never	Factors associated with increased risk for female papillary cancer were low intake of cruciferous vegetables and seafood.	Papillary: 71; mixed (papillary/follicular): 33	8
#36	Chatenoud et al. (35)	Italy	Case-control	428/3526	<75	Cases: 116/312; controls: 2069/1457	Refined-cereal intake: the highest tertile vs. the lowest tertile	Consumption of refined cereals was associated with an increased risk of cancers of the large bowel, the stomach, and other selected digestive and non-digestive sites.	Not mentioned	8
#37	Liang et al. (5)	America	Case-control	390/436	21–84	Cases: 71/319; controls: 128/309	Starchy foods and desserts: the quartile (lowest quartile as the reference group). Fruits and vegetables: the quartile (lowest quartile as the reference group). High protein and fat: the quartile (lowest quartile as the reference group).	1. A significant negative association between diet patterns rich in fruits and vegetables and TC risk, especially among women aged 50 years or older. 2. While high in starchy foods and desserts may be positively and negatively associated with TC risk among men and women, respectively	Papillary: 329; follicular: 50; medullary: 9	8

TC, thyroid cancer; DTC, differentiated thyroid cancer; BMI, body mass index; PCB, polychlorinated biphenyl.

### 2.4 Quality assessment

The quality of the included studies was assessed using the New Castries-Ottawa Scale (NOS). NOS evaluates the quality of the literature using the semi-quantitative principles, including selection, comparability, and exposure/outcome. Furthermore, study quality appraisal (out of 9 points) was divided into three groups: high-quality evidence (≥7 points), moderate-quality evidence (5–6 points), and low-quality evidence (4 points). If there was disagreement about the rating of a study, all authors researched the article and came to a consensus.

### 2.5 Statistical analysis for meta-analysis

Odds ratios (ORs), hazard ratios (HRs) or risk ratios (RRs), and 95% confidence intervals (CIs) were extracted and merged using STATA software (v.17.0) and Review Manager (v.5.4). Random-effects model was used for all studies. Quantitative analysis of inter-study heterogeneity was assessed using Cochran’s Q test and I^2^ statistic. *P*-values < 0.1 were considered indicative of heterogeneity for the chi-squared test. Heterogeneity was classified as low if I^2^ < 30%, moderate if I^2^ = 30%–60% and high if I^2^ > 60% (Supplementary Table 1). Publication bias was assessed through funnel plot, Begg’s rank correlation, and Egger’s weighted regression tests (Supplementary Table 2). When there was evidence of funnel plot asymmetry, potentially missing studies were imputed using the trim and fill method. Additional sensitivity analysis was performed using the Cochrane Handbook for Systematic Reviews of Interventions (V.6.1, 2020). For subgroup analysis, we regarded the lowest quantile in the study as low intake and the highest quantile as high intake, and conducted a pooled analysis of the relationship between high and low intake of various foods and thyroid cancer. We used Hill’s causal criteria to assess the possibility of a protective or enhanced causal relationship between high intake of various diets and thyroid cancer (Supplementary Table 3) (12).

### 2.6 Data sources for MR analysis

**FIGURE 1:**
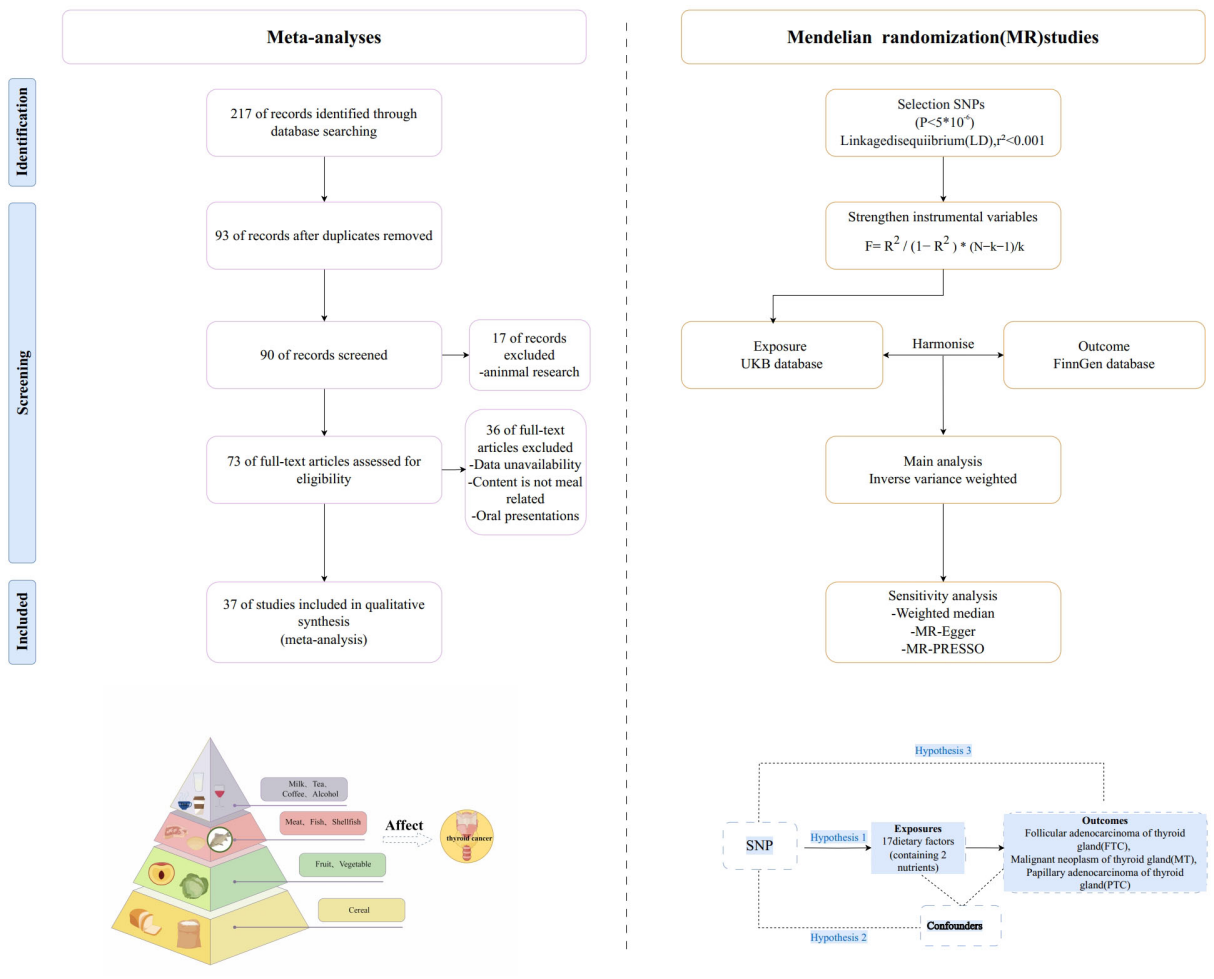
Flow diagram for identification of studies and three fundamental assumptions of a MR analysis.

We selected 377 dietary factors (containing 37 nutrients) associated with thyroid cancer as exposure data and summary level data from the UK Biobank (as of August 1, 2024), including the major food groups of cereal, fruits and vegetables, meat and fish (shellfish), milk and eggs, and beverages (alcohol, tea, etc.). Thyroid cancer is a malignant tumor that originates from the follicular epithelial cells or parafollicular epithelial cells of the thyroid gland (13). Outcome data selected three thyroid malignancies from the FinnGen biological sample library, namely follicular adenocarcinoma of thyroid gland (FTC), malignant neoplasm of thyroid gland (MT), papillary adenocarcinoma of thyroid gland (PTC). Because of the re-analysis of previously summarized data, no additional ethical approval was required. Supplementary Tables 4, 5 provides a comprehensive description of the data sources for the respective result datasets. MR relies on three fundamental hypotheses: (1) The IVs hypothesis: The genetic variant selected as the IVs is unintentionally related to the exposure of interest in a casual manner. (2) The used genetic variants should not be associated with potential confounding variables in the exposure-outcome relationship. (3) The Pleiotropy Hypothesis: The genetic variant used as the IV is associated solely with the outcome via its effect on the exposure and no other biological pathways (Figure 1). A quality assessment was conducted based on adherence to the Strengthening the Reporting of Mendelian Randomization Studies (STROBE-MR) Guidelines (Supplementary File 2).

### 2.7 SNP selection and strengthen IVs

We identified SNPs closely related to exposure and outcome, to ensure that the number of SNPs shared between exposure and outcome is sufficient, with genome-wide significance (*P* < 5 × 10^–6^). These selected SNPs are located in different gene regions and do not exhibit significant linkage disequilibrium (r^2^ < 0.001). Supplementary Table 6 contains additional detailed information related to the IVs used in our study. To minimize the impact of weak IVs on the causal analysis, F-statistic was used. The value greater than 10 indicates a low probability of weak instrument bias, indicating that the IVs possess sufficient strength to generate reliable and unbiased causal estimates in MR analysis. So we exclude all the tools where the F-statistic is less than 10 (e.g., 19 SNPs used for satsuma intake, all with F-statistics > 10). Evaluating of weak instrument bias helps ensure the validity and robustness of our findings related to the causal relationships between the exposure (dietary factors) and the outcomes (thyroid cancers).

### 2.8 Statistical analysis for MR analysis

The inverse variance weighting (IVW), MR-Egger, and weighted median (WM) methods were used to examine a causal association, with IVW being the primary analytical method. Horizontal pleiotropy is a main source of bias in MR, whereby genetic variants influence the exposure and outcome via two separate biological pathways. The IVW method can achieve unbiased causal estimates without horizontal pleiotropy where the variants affect the direction and outcome through pathways that are not on the causal pathway of interest. Diets affect disease only when two or more statistical methods are in the same direction. If only the IVW approach yields support for the influence of a factor, and the other approaches are consistent in the direction of the beta, based on previous research. We consider this factor to be a potential influencing factor. Several sensitivity analysis were conducted to obtain stable MR estimates. The IVW and MR-Egger were utilized to quantify the heterogeneity effect among genetic instruments. Cochran’s Q test assessed heterogeneity in the IVW model. Cochran’s Q test *P* < 0.05 indicates the presence of heterogeneity. Finally, the leave-one-out method was utilized to address sensitivity analysis. All analyses were performed in R software (version 4.2.1) using the TwoSampleMR package.

## 3 Results

### 3.1 Meta-analysis

#### 3.1.1 Search results

The results of our initial screening by combining search records from all databases showed that 3 duplicate studies were removed. The 90 studies were screened from the titles and abstracts, and after careful evaluation of the full text, thirty-seven studies (including 16 cohort studies and 21 case-control studies) were ultimately included in the systematic review and meta-analysis (Figure 1).

#### 3.1.2 Study characteristics and quality assessment

These studies evaluated the association of thyroid cancer risk with the risk of specific food groups such as vegetables (*n* = 7) and fruits (*n* = 8), meat (*n* = 5), refined grains (*n* = 2), all fish (*n* = 9), milk (*n* = 6), starchy foods (*n* = 4), freshwater fish (*n* = 4), alcohol (*n* = 6), shellfish (*n* = 8), saltwater fish (*n* = 4), seaweed (*n* = 3), coffee (*n* = 5), and tea (*n* = 4). Of all the included studies, most of them were conducted in the United States and Korea. The quality assessment scores for case-control and cohort studies ranged from 6 to 9 (Table 1).

#### 3.1.3 Meta-analysis result of different food groups

##### 3.1.3.1 Refined cereal and starchy foods consumption

**FIGURE 2:**
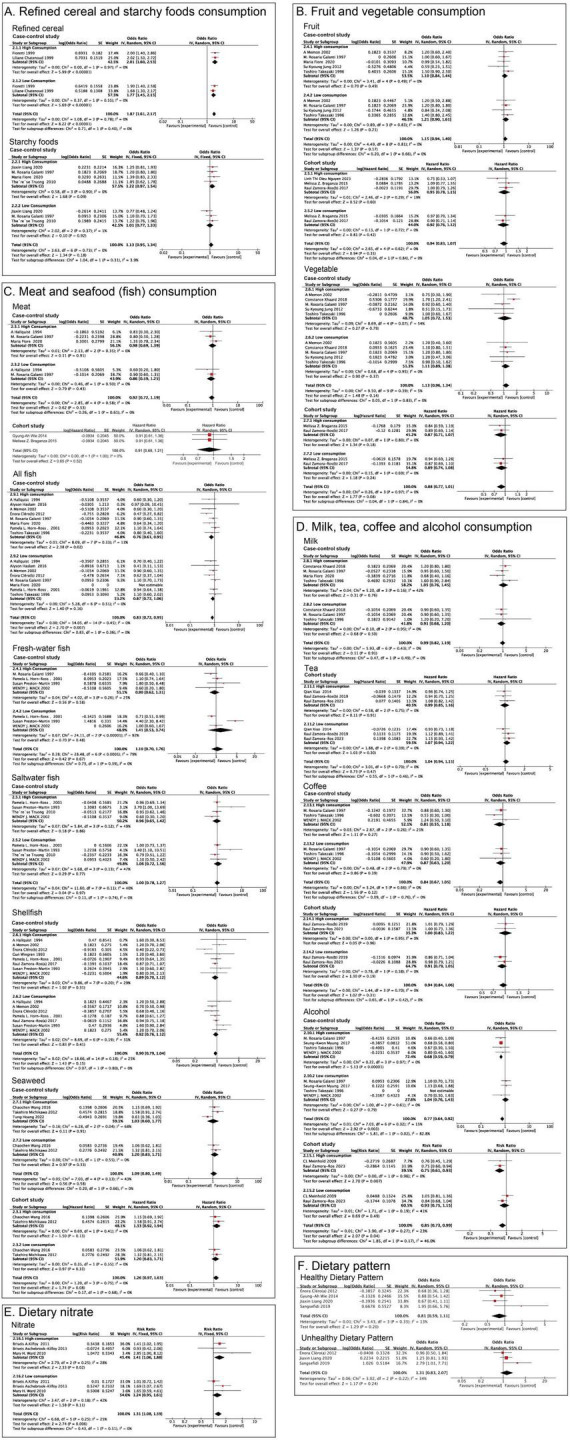
Meta-analysis for associations of the food groups and dietary pattern with thyroid cancer risk. **(A)** Refined cereal and Starchy foods; **(B)** Fruit and Vegetable; **(C)** Meat, All fish, Fresh-water fish, Saltwater fish, Shellfish and Seaweed; **(D)** Milk, Tea, Coffee and Alcohol; **(E)** Nitrate; **(F)** Healthy Dietary Pattern and Unhealthy Dietary Pattern.

Two case-control studies in the literature were used to examine the relationship between refined grain consumption and thyroid cancer risk. The pooled results indicated that high consumption of refined grains increased the risk of thyroid cancer with low heterogeneity (*P* < 0.00001, OR = 2.01, 95% CI = 1.60–2.53, I^2^ = 0%, Figure 2A and Supplementary Table 1). Subgroup analysis showed that consuming a smaller amount of refined grains still increased the risk of thyroid cancer (*P* < 0.00001, OR = 1.77, 95% CI = 1.45–2.15, I^2^ = 0%, Figure 2A and Supplementary Table 1). Four case-control studies examined the relationship between starchy food consumption and thyroid cancer risk. No statistical differences were observed in the pooled results (*P* = 0.09, OR = 1.22, 95% CI = 0.97–1.54, I^2^ = 0%, Figure 2A and Supplementary Table 1). After aggregating the results of consuming less starchy foods, there was still no statistical difference (*P* = 0.92, OR = 1.01, 95% CI = 0.77–1.33, I^2^ = 1%, Figure 2A and Supplementary Table 1).

##### 3.1.3.2 Fruit and vegetable consumption

Three cohort studies and five case-control studies assessed fruit consumption and thyroid cancer risk. There was no significant association between more fruit consumption and thyroid cancer in the case-control (*P* = 0.49, OR = 1.10, 95% CI = 0.84–1.44, I^2^ = 0%) and cohort studies (*P* = 0.60, HR = 0.95, 95% CI = 0.78–1.15, I^2^ = 19%, Figure 2B and Supplementary Table 1). Similarly, whether in case-control studies (*P* = 0.21, OR = 1.21, 95% CI = 0.90–1.61, I^2^ = 0%) or cohort studies, consuming smaller amounts of fruit is also not associated with thyroid cancer (*P* = 0.42, HR = 0.92, 95% CI = 0.76–1.12, I^2^ = 0%, Figure 2B and Supplementary Table 1). There were no statistically significant differences between vegetables and thyroid cancer (case-control: *P* = 0.79, OR = 1.05, 95% CI = 0.72–1.53, I^2^ = 54%; cohort: *P* = 0.18, RR = 0.87, 95% CI = 0.71–1.07, I^2^ = 0%, Figure 2B and Supplementary Table 1). After subgroup analysis, a lower intake of vegetables was also not associated with the risk of thyroid cancer (case-control: *P* = 0.37, OR = 1.11, 95% CI = 0.89–1.38, I^2^ = 0%; cohort: *P* = 0.24, RR = 0.89, 95% CI = 0.74–1.08, I^2^ = 0%, Figure 2B and Supplementary Table 1).

##### 3.1.3.3 Meat and seafood (fish) consumption

Two cohort and three case-control studies explored the relationship between total meat consumption and thyroid cancer risk. The pooled results showed that high meat intake was not statistically different from the risk of thyroid cancer (case-control: *P* = 0.91, OR = 0.98, 95% CI = 0.69–1.39, I^2^ = 6%; cohort: *P* = 0.52, HR = 0.91, 95% CI = 0.69–1.21, I^2^ = 0%, Figure 2C and Supplementary Table 1). In case-control studies, there was no statistically significant difference between low meat intake and the risk of thyroid cancer. Cohort studies did not present data on low intake (case-control: *P* = 0.43, OR = 0.86, 95% CI = 0.59–1.25, I^2^ = 0%, Figure 2C and Supplementary Table 1).

All fish consumption was associated with reduced risk of thyroid cancer risk (*P* = 0.02, OR = 0.76, 95% CI = 0.61–0.95, *I^2^* = 13%, Figure 2C and Supplementary Table 1). In the analysis of all high-consumption and low-consumption subgroups of fish, since Maria Fiore 2020 did not provide data on low-consumption, the remaining data combined showed that all fish were not associated with thyroid cancer at lower intake levels (*P* = 0.16, OR = 0.87, 95% CI = 0.72–1.06, I^2^ = 0%, Figure 2C). However, no statistically significant differences in outcomes were observed in high consumption of freshwater fish (*P* = 0.58, OR = 0.90, 95% CI = 0.62–1.31, I^2^ = 25%), saltwater fish consumption (*P* = 0.86, OR = 0.96, 95% CI = 0.65–1.42, I^2^ = 49%), shellfish consumption (*P* = 0.31, OR = 0.89, 95% CI = 0.70–1.12, I^2^ = 29%), seaweed consumption (case-control: *P* = 0.91, OR = 1.03, 95% CI = 0.60–1.77, I^2^ = 68%; cohort: *P* = 0.13, HR = 1.33, 95% CI = 0.92–1.94, I^2^ = 0%, Figure 2C and Supplementary Table 1). Subgroup analysis showed that low-consumption freshwater fish (*P* = 0.48, OR = 1.41, 95% CI = 0.53–3.74, I^2^ = 92%), saltwater fish (*P* = 0.77, OR = 1.06, 95% CI = 0.72–1.56, I^2^ = 47%), and shellfish (*P* = 0.41, OR = 0.92, 95% CI = 0.76–1.12, I^2^ = 31%), seaweed consumption (case-control: *P* = 0.33, OR = 1.20, 95% CI = 0.83–1.71, I^2^ = 0%; Cohort: *P* = 0.33, HR = 1.20, 95% CI = 0.83–1.71, I^2^ = 0%). There is no statistically significant difference in the results of Figure 2C and Supplementary Table 1. However, when high and low consumption volumes are combined, there is a certain degree of heterogeneity (fresh-water fish: I^2^ = 79%; saltwater fish: I^2^ = 40%; shellfish: I^2^ = 25%).

##### 3.1.3.4 Milk, tea, coffee and alcohol consumption

There were no statistical differences in consumption of milk (*P* = 0.76, OR = 1.05, 95% CI = 0.76–1.45, I^2^ = 42%), tea (*P* = 0.91, HR = 0.99, 95% CI = 0.85–1.16, I^2^ = 0%), coffee (Case-control: *P* = 0.27, OR = 0.81, 95% CI = 0.55–1.18, I^2^ = 25%; Cohort: *P* = 0.96, HR = 1.0, 95% CI = 0.83–1.22, I^2^ = 0%, Figure 2D and Supplementary Table 1). Drinking less milk (*P* = 0.50, OR = 0.91, 95% CI = 0.68–1.20, I^2^ = 0%), tea (*P* = 0.30, HR = 1.07, 95% CI = 0.94–1.22, I^2^ = 0%), and coffee (Case-control: *P* = 0.39, OR = 0.87, 95% CI = 0.63–1.20, I^2^ = 0%; Cohort: *P* = 0.19, HR = 0.91, 95% CI = 0.79–1.05, I^2^ = 0%, Figure 2D and Supplementary Table 1) also showed no statistical difference. A total of four case-control studies and two cohort studies examined the relationship between total alcohol consumption and thyroid cancer risk. The pooled results of both the case-control studies and the cohort studies indicated that alcohol was a protective factor against thyroid cancer and reduced the risk of thyroid cancer (Case-control: *P* < 0.00001, OR = 0.68, 95% CI = 0.59–0.79, I^2^ = 0%, Cohort: *P* = 0.007, RR = 0.75, 95% CI = 0.61–0.93, I^2^ = 0%, Figure 2D and Supplementary Table 1). However, subgroup analyses showed that a small amount of alcohol intake was not associated with thyroid cancer, whether in case-control studies or cohort studies (Case-control: *P* = 0.79, OR = 1.04, 95% CI = 0.76–1.43, I^2^ = 0%, Cohort: *P* = 0.49, RR = 0.93, 95% CI = 0.75–1.15, I^2^ = 41%, Figure 2D and Supplementary Table 1).

##### 3.1.3.5 Nitrate intake consumption

High consumption of dietary nitrate intake increased thyroid cancer risk and some heterogeneity was observed (*P* = 0.02, RR = 1.41, 95% CI = 1.06–1.88, I^2^ = 28%, Figure 2E and Supplementary Table 1). The results of subgroup analysis showed that consuming a small amount of nitrate was not associated with thyroid cancer and was no longer a risk factor for thyroid cancer (*P* = 0.11, RR = 1.24, 95% CI = 0.95–1.61, I^2^ = 42%, Figure 2E and Supplementary Table 1).

##### 3.1.3.6 Dietary pattern

Healthy dietary patterns (Balanced diets, traditional Bosnian diets) may reduce thyroid cancer risk, and unhealthy dietary patterns (Meat and western dietary patterns) may elevate thyroid cancer risk, but neither is significantly different (Healthy diet: *P* = 0.20, OR = 0.81, 95% CI = 0.59–1.11, I^2^ = 13%, Unhealthy diet: *P* = 0.24, OR = 1.31, 95% CI = 0.83–2.07, I^2^ = 34%, Figure 2F and Supplementary Table 1).

#### 3.1.4 Public bias

This meta-analysis showed no publication bias for studies with the following correlations, and the asymmetry of the funnel plot was confirmed by Egger and Begg tests (Supplementary Figure 1 and Supplementary Table 2).

### 3.2 MR analysis

**FIGURE 3:**
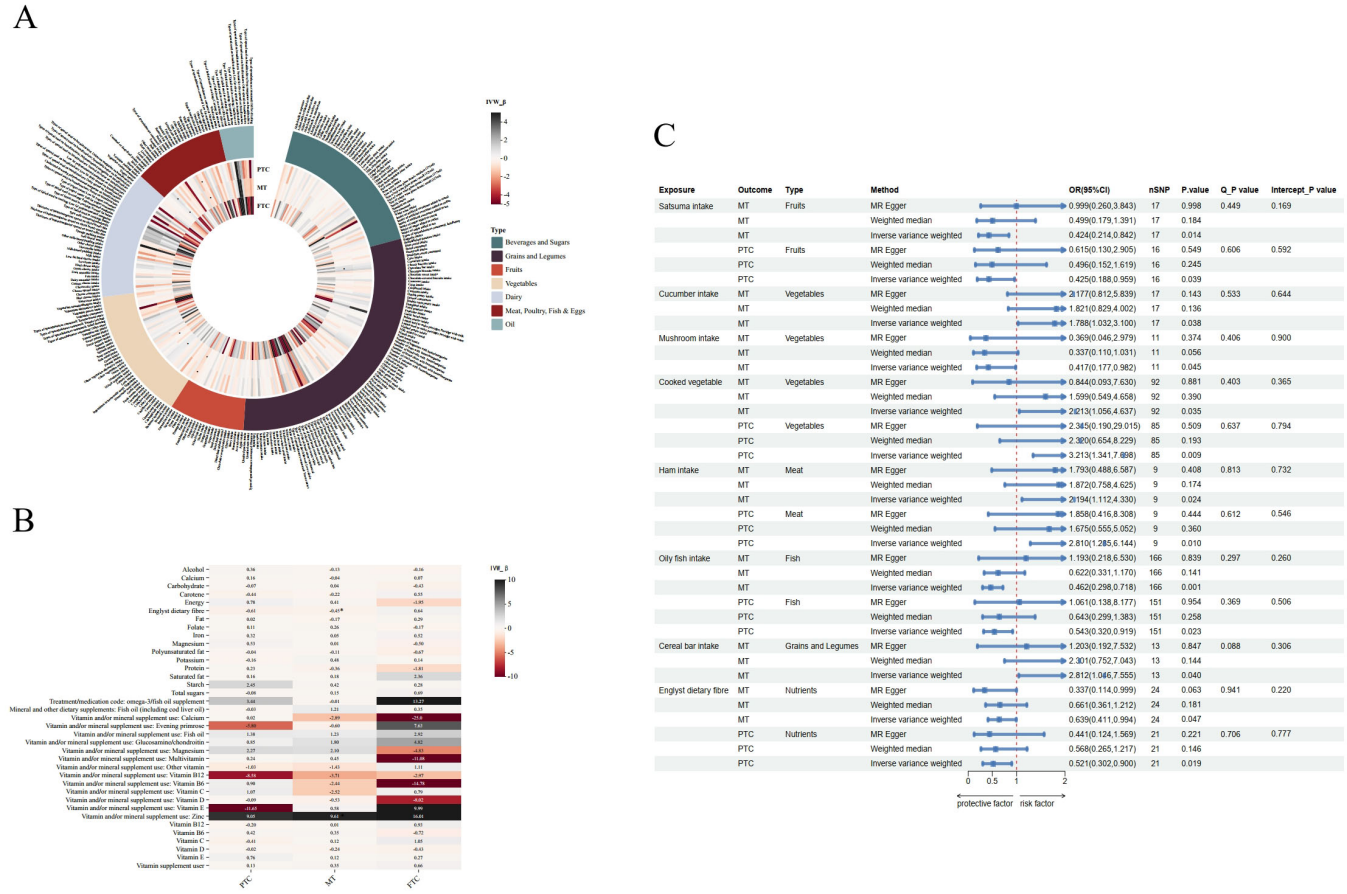
Mendelian randomization (MR) analysis for the causal associations of dietary factors and thyroid cancer risk. **(A)** The heatmap shows the beta values of the IVW method, *P < 0.05. **(B)** Causal risk between food and three types of thyroid cancer was estimated using conventional IVW MR analysis, MR-Egger, and weighted median MR. **(C)** Forest plot and sensitivity analysis of MR analysis showing the effect of food on the risk of thyroid cancer.

The baseline characteristics of the 345,313 (FTC), 347,429 (MT) and 346,859 (PTC) eligible participants were shown in Table 1. Figures 3A, B illustrate the beta values for various food intakes or nutrients in three types of thyroid cancer MR analysis with IVW methods. A positive beta value indicates a significant association between the exposure and outcome, with higher values representing a greater effect size. Figure 3C shows the estimated causal effects of different foods/nutrients on thyroid cancer, as well as a forest plot of the estimated values for each outcome using different MR methods (see detailed results in Supplementary Tables 7, 8).

#### 3.2.1 Cereal

Cereal intake and thyroid cancer studies found that cereal bars showed a positive causal relationship with MT (OR: 2.812, 95% CI: 1.046–7.555, *P* = 0.040).

#### 3.2.2 Fruit and vegetable

In a study examining the relationship between fruit intake and thyroid cancer, the results showed a statistically significant correlation between satsuma consumption and an decreased risk of MT and PTC. The OR for satsuma intake in IVW analysis was 0.424 (95% CI: 0.214–0.842; *P* = 0.014) in MT, and was 0.425 (95% CI: 0.188–0.959; *P* = 0.039) in PTC. Regarding vegetable intake and thyroid cancer, cucumber consumption demonstrated a positive causal relationship with MT, as seen in the IVW analysis with an OR of 1.788 (95% CI: 1.032–3.100; *P* = 0.038). Additionally, cooked vegetable intake showed a positive causal relationship in MT (OR: 2.213, 95% CI: 1.056–4.637; *P* = 0.035) and PTC (OR: 3.213, 95% CI: 1.341–7.698; *P* = 0.009). In contrast, mushroom intake demonstrated a negative causal relationship with MT, as seen in the IVW analysis with an OR of 0.417 (95% CI: 0.177–0.982; *P* = 0.045).

#### 3.2.3 Meat, fish and shellfish

In meat intake and thyroid cancer, ham consumption showed a positive causal relationship with MT (OR: 2.194; 95% CI: 1.112–4.330; *P* = 0.024) and PTC (OR: 2.810, 95% CI: 1.285–6.144; *P* = 0.010). In fish or shellfish intake and thyroid cancer, oily fish consumption demonstrated a significant negative causal relationship with MT (OR: 0.462, 95% CI: 0.298–0.718, *P* = 0.001). Oily fish also showed consistent results in PTC (OR: 0.543, 95% CI: 0.320–0.919, *P* = 0.023), while no association has been found between shellfish consumption and thyroid cancer.

#### 3.2.4 Englyst dietary fiber

We identified Englyst dietary fiber as nutrients of interest. The study found that Englyst dietary fiber intake was negatively associated with MT (OR: 0.639, 95% CI: 0.411–0.994; *P* = 0.047) with no heterogeneity or horizontal pleiotropy. Similarly, Englyst dietary fiber showed a negative causal relationship with PTC (OR: 0.521, 95% CI: 0.302–0.900; *P* = 0.019).

#### 3.2.5 Sensitivity analysis

The sensitivity analysis findings are presented in Figure 3C. Cochran’s Q test shows that there is no heterogeneity in our results. The findings of the MR-Egger intercept test revealed no horizontal pleiotropy (*P* all > 0.05). The results were not significantly changed before or after MR-PRESSO correction for outliers. The Steiger directionality test also shows that there is no reverse causality in our analyses, as detailed in Supplementary Table 9. Supplementary Figures 2, 3 illustrate the scatter plots and forest plots of the MR analysis. The leave-one-out analyses indicated that no individual SNP had a significant impact on the MR estimate results, as the OR values were all on the one side of the zero-line. The comparison of results from the meta-analysis and Mendelian randomization (MR) analyses has been included in Supplementary Table 10.

## 4 Discussion

This comprehensive study investigated the relationships between dietary factors and thyroid cancer risk, firstly provided comparisons between observational associations by meta-analysis and genetically estimated causality by MR analyses. The meta-analysis is based on case-control studies and cohort studies examining the relationship between specific food groups and dietary patterns and the risk of thyroid cancer. MR analysis further revealed the potential causal effects of specific foods.

When assessing the causal relationship between thyroid cancer risk and dietary factors, this study emphasized temporality as a necessary condition for causal inference based on the Hill criterion. All the included studies conformed to the temporality of the Hill causal relationship. Meta-analysis shows that the intake of refined cereal (OR = 2.01) and nitrates (OR = 1.41) is significantly positively correlated with the risk of thyroid cancer. The mechanism involves hyperinsulinemia and elevated insulin-like growth factor I caused by refined carbohydrates (14), and nitrate is converted into carcinogenic N-nitroso compounds in the body and competitively inhibits iodine absorption (15–17). Conversely, high consumption of fish (OR = 0.91) and alcohol (OR = 0.68) showed protective effects, which might be related to the alleviation of thyroid-related dysfunction by omega-3 polyunsaturated fatty acids in fish and the influence of alcohol on the hypothalamic-pituitary-thyroid axis function (18–20). Among them, the intake of fish also presented a dose-response relationship. The correlations of the other factors were not significant. Consistency assessment indicates that there are inconsistencies among some studies regarding the relationship between nitrate removal and thyroid cancer, while other significant factors all meet the Hill consistency criteria. For the experimental evidence in Hill causality, since randomized controlled trials were not included in this meta-analysis, the NOS scale was used to evaluate the quality of the studies. Except for Hoang et al., 2022 (21) and Mack et al., 2002 (22), the scores of other articles were all 7 points or above, which belonged to high-quality studies.

According to common perception, consuming fruits and vegetables is believed to have a protective effect against cancer. A previous study suggested that high consumption of raw vegetables, persimmons, and oranges may lower the risk of developing thyroid cancer and even help prevent early-stage thyroid cancer (23). Consumption of oranges was consistent with our MR findings, this may be because citrus fruits are a good source of bioactive compounds such as flavonoids, vitamin C, carotenoids, limonene and citric acid. Citrus flavonoids are known to have strong anti-proliferative activity in human cancer cells (24). The β-glucans and polysaccharides in mushrooms can activate immune cells, inhibit tumor growth, and show anticancer potential by regulating a single molecule in specific signaling pathways, or by having multiple targets in the same or different signaling pathways (including PI3K/Akt, Wnt/β-catenin and MAPK pathways) (25). Cooked vegetables seem to demonstrate hazardous effects on thyroid cancer. Overcooking may destroy antioxidant components such as vitamin C, while the reduced saponins in cruciferous vegetables (which are goitrogenic factors) still pose potential interference with iodine metabolism through thiocyanate accumulation. These compounds competitively inhibit thyroid iodine uptake and exhibit strong heat resistance (26, 27). However, current MR analysis did not account for iodine intake, potentially amplifying the risks associated with cooked vegetables. Cucumber has been shown to have a risk effect on thyroid cancer, but the existing literature does not support this conclusion. We consider that it is related to the cooking and eating methods of cucumber (raw with skin or peeled and cooked). For example, pickled cucumber contains a large amount of salt and nitrites, which may promote the enlargement of thyroid hormones or form carcinogenic nitrosamines, and indirectly increase the risk of thyroid cancer (28). While research on the relationship between meat intake and thyroid cancer risk is limited, and in their study, Tavani et al. identified red meat intake as an important factor in the nutritional etiology of cancer in humans (29). Only ham was associated with the risk of thyroid cancer in MR. And Ham intake data was from questionnaires “How many slices of ham, Parma ham, salami, pastrami, cured meats did you have?” Ham appears to be a risk factor, potentially due to the high nitrate content in ham. Another study of dietary patterns and differentiated thyroid cancer found that a diet of low-fat meats may be an important protective factor and thus further breakdown of meat types or different types of meat processing needs to be studied (30).

A previous study stated that higher intake of milk and dairy products can prevent thyroid cancer (4). While our analysis did not find a clear relationship between milk intake and thyroid cancer. Another case-control study in Sweden and Norway also found no association between milk consumption and thyroid cancer (31).

Nitrates inhibit the uptake of iodide by the thyroid gland, decreasing levels of T3 and T4, which increase thyroid-stimulating hormones (16). Some studies have observed that increased dietary nitrate intake is associated with an increased risk of thyroid cancer (32), and our findings also show that high nitrate intake is a risk factor for thyroid cancer. Polyphenols such as flavonoids and phenolic acids, which are abundant in tea, may play a role in thyroid cancer by regulating enzymatic activities and signaling pathways related to cell proliferation, differentiation, apoptosis, and inflammation (33). The EPIC study found no association between tea intake and thyroid cancer risk (34), which is consistent with our findings.

Previous researches found an increased risk of thyroid cancer has been shown to be associated with excessive intake of starch-rich foods such as refined grains, pasta or rice, bread, pastries and potatoes (35, 36). In terms of plasma glucose and insulin responses, GI and GL are indicators of physiological responses to different foods and are highly correlated with high intake of refined carbohydrates (37). High dietary levels of GI and GL are associated with thyroid cancer risk, while high total energy may increase the risk of differentiated thyroid cancer (38). This is similar to our results, but we did not find significant differences in the pooled starchy foods, which may be due to their different foods and therefore different levels of GI after starch digestion. Caffeine in coffee increases intracellular levels of cyclic adenosine monophosphate, which has an inhibitory effect on tumor growth (39). Meanwhile, chlorogenic acid in coffee is an antioxidant, and its activity is thought to play a role in preventing cancer (40). However, we did not find a link between coffee and thyroid cancer, suggesting that more prospective studies may be needed to examine the impact of coffee consumption on thyroid cancer risk, in line with previous conclusions (41).

Since different foods are often consumed in combination and interact with each other in complex situations in the daily diet, a more comprehensive dietary pattern provides a constructive tool for assessing the impact of diet on health (42). This meta-analysis harmonized balanced diets, traditional Bosnian diets and healthy diets as healthy dietary patterns. Meat, high fat intake and Western dietary patterns were harmonized as unhealthy dietary patterns. Previous studies have shown that a healthy dietary pattern may reduce the risk of cardiovascular disease and total mortality (43), and it has also been associated with a lower risk of several cancers (44, 45). In contrast, unhealthy dietary patterns such as the Western dietary pattern have been associated with an increased chance of developing differentiated thyroid cancer (46). However, the pooled results of this meta-analysis showed that there was no statistically significant difference between the different dietary patterns and the risk of thyroid cancer, which may be related to the small number of included literature and some heterogeneity, and further studies are recommended to provide more conclusive evidence about the association between dietary patterns and thyroid cancer.

Our MR results indicated that oily fish consumption both decrease the risk of developing thyroid cancer. Prior research has shown that moderate fish consumption does not significantly elevate the risk of thyroid cancer, and may even have a beneficial effect in areas with iodine deficiency (47), which is relatively consistent with our findings. In the study of thyroid cancer in women, Mack et al. found that shellfish and saltwater fish reduced risk independent of the risk of fish consumption, but our meta-study found no significant relationship between high shellfish and saltwater fish consumption and thyroid cancer risk (22). One possible explanation is that iodine deficiency or excess may exist in the study area. In areas of severe iodine deficiency, a high intake of fish had a protective effect, whereas in areas of adequate iodine intake there was no effect (48).

This meta-analysis has some limitations, firstly the small number of studies pooled and combined in some food groups does not accurately reflect the true picture. Secondly, there was some variation in consumption categories among the included studies, and different exposure assessment methods can cause some bias. The data on fish research was included after a comprehensive search of published literature. However, only two studies included more than 1,000 people, while the rest were small-scale research results. More large-scale studies are needed in the future to confirm our findings. However, there are some advantages, firstly, we observed inconsistent results across different study designs, so different study designs were pooled and aggregated separately. Second, the literature search was conducted independently by other authors to minimize errors. We employed the MR design to reduce the risks of confounding and reverse causation, which are significant limitations of observational studies. However, there are some limitations associated with MR as well. Although we selected the largest sample size and the most recent GWAS datasets available for our MR analysis, the sample size and number of events in our study were relatively small compared to those in population-based studies. Due to the generally low heritability of dietary exposure, which may limit statistical efficacy, future studies need to combine high-precision metabolomics or proteomics data to improve the explanatory power of instrumental variables. This study mainly explores the long-term average effect of dietary exposure. In the future, potential threshold effects can be explored by stratified MR analysis (e.g., stratified by intake) or non-linear MR methods (e.g., quadratic model), which require a larger sample size to support. Additionally, our analysis was performed on populations with specific ancestries, which may introduce ascertainment bias. Consequently, these findings may not accurately reflect the broader population. Furthermore, the lack of demographic and detailed clinical information in the GWAS database hindered our ability to conduct subgroup analysis, limiting the depth of our insights.

## 5 Conclusion

This study conducted a meta-analysis of case-control and cohort studies to summarize the evidence for the association between specific food groups, dietary patterns, and thyroid cancer risk. We also conducted an MR analysis to investigate the relationship between specific foods and thyroid cancer. Both meta-analysis and MR analysis found that fish was associated with a reduced risk of thyroid cancer.

## Data Availability

The datasets presented in this study can be found in online repositories. The names of the repository/repositories and accession number(s) can be found in the article/Supplementary material.

## Supplementary material

Supplementary Figure 1 Funnel plot detailing publication bias in the case-control and cohort studies reporting.

Supplementary Figure 2 The scatter plot for MR analysis of the causal relationship between different dietary factors SNPs and three types of thyroid cancer.

Supplementary Figure 3 Results of leave-one-out sensitivity analysis.
