# Supplementary material for: Dissecting the causal association of diet with thyroid cancer: a systematic review with meta-analysis and mendelian randomization analysis
Source: Front Nutr. 2025 Sep 17;12:1664129. doi: 10.3389/fnut.2025.1664129 (PMC12486595; doi:10.3389/fnut.2025.1664129)
Supplement: Supplementary file 2 [file Supplementary_file_2.docx]

**Additional File 1.**

**Table S1.** STROBE-MR checklist of recommended items to address in reports of Mendelian randomization studies.

| Item No. | Section | Checklist item | Page No. | Relevant text from manuscript |
| --- | --- | --- | --- | --- |
| 1 | TITLE and ABSTRACT | Indicate Mendelian randomization (MR) as the study’s design in the title and/or the abstract if that is a main purpose of the study | 1 | - Title:Dissecting the causal association of diet with thyroid cancer risk: A systematic review with meta-analysis and mendelian randomization analysis.  - Abstract:Additionally, we conducted mendelian randomization analysis by selecting dietary factors (including nutrients) associated with thyroid cancer as exposure data to complement the results of meta-analysis. |
|  | INTRODUCTION |  |  |  |
| 2 | Background | Explain the scientific background and rationale for the reported study. What is the exposure? Is a potential causal relationship between exposure and outcome plausible? Justify why MR is a helpful method to address the study question | 2 | - Dietary factors are considered putative risk factors for the development of cancer at different sites, while nutritional factors play an important role in the pathogenesis of different metabolic diseases.  - Besides, mendelian randomization (MR) uses genetic variants as instrumental variables (IVs) to estimate the causal effect of an exposure (diet) on an outcome (thyroid cancer) |
| 3 | Objectives | State specific objectives clearly, including pre-specified causal hypotheses (if any). State that MR is a method that, under specific assumptions, intends to estimate causal effects | 2 | The main objective of this meta-analysis is to outline the relationship between food groups (vegetables, fruits, meat, dairy products, tea, alcohol, seafood [fish, saltwater fish, shellfish and fresh-water fish]), dietary patterns and thyroid cancer, then MR was used to complement the observational findings. which will help in the development and prevention of effective strategies for thyroid cancer. |
|  | METHODS |  |  |  |
| 4 | Study design and data sources | Present key elements of the study design early in the article. Consider including a table listing sources of data for all phases of the study. For each data source contributing to the analysis, describe the following: |  |  |
|  | a) | Setting: Describe the study design and the underlying population, if possible. Describe the setting, locations, and relevant dates, including periods of recruitment, exposure, follow-up, and data collection, when available. | 3 | See Figure 1 for a schematic summary of the study design.  Exposure: Supplementary Table 4.  Outcome: Supplementary Table 4. |
|  | b) | Participants: Give the eligibility criteria, and the sources and methods of selection of participants. Report the sample size, and whether any power or sample size calculations were carried out prior to the main analysis | 3 | Exposure: Supplementary Table 4.  Outcome:Supplementary Table 4.  (A quality assessment was conducted based on adherence to the Strengthening the Reporting of Mendelian Randomization Studies (STROBE-MR) Guidelines (Supplementary file 2)) |
|  | c) | Describe measurement, quality control and selection of genetic variants | 3 | - We identified SNPs closely related to exposure and outcome, with genome-wide significance ( P<5 * 10-6). These selected SNPs are located in different gene regions and do not exhibit significant linkage disequilibrium (r2<0.001). Supplementary Table S4 contains additional detailed information related to the instrumental variables used in our study.  - To minimize the impact of weak IVs on the causal analysis, F-statistic was used. The value greater than 10 indicates a low probability of weak instrument bias, indicating that the IVs possess sufficient strength to generate reliable and unbiased causal estimates in MR analysis. |
|  | d) | For each exposure, outcome, and other relevant variables, describe methods of assessment and diagnostic criteria for diseases | 3 | Thyroid cancer is a malignant tumor that originates from the follicular epithelial cells or parafollicular epithelial cells of the thyroid gland. |
|  | e) | Provide details of ethics committee approval and participant informed consent, if relevant |  | Because of the reanalysis of previously summarized data, no additional ethical  approval was required. |
| 5 | Assumptions | Explicitly state the three core IV assumptions for the main analysis (relevance, independence and exclusion restriction) as well assumptions for any additional or sensitivity analysis | 3 | Mendelian randomization relies on three fundamental hypotheses:1) The Instrumental Variable Hypothesis: The genetic variant selected as the instrumental variable (IV) is unintentionally related to the exposure of interest in a casual manner. 2) The used genetic variants should not be associated with potential confounding variables in the exposure–outcome relationship. 3) The Pleiotropy Hypothesis: The genetic variant used as the IV is associated solely with the outcome via its effect on the exposure and no other biological pathways. Figure 1 depicts the MR design’s flowchart. |
| 6 | Statistical methods: main analysis | Describe statistical methods and statistics used |  |  |
|  | a) | Describe how quantitative variables were handled in the analyses (i.e., scale, units, model) | NA |  |
|  | b) | Describe how genetic variants were handled in the analyses and, if applicable, how their weights were selected | NA |  |
|  | c) | Describe the MR estimator (e.g. two-stage least squares, Wald ratio) and related statistics. Detail the included covariates and, in case of two-sample MR, whether the same covariate set was used for adjustment in the two samples | 3 | IVW method can achieve unbiased causal estimates without horizontal pleiotropy where the variants affect the direction and outcome through pathways that are not on the causal pathway of Interest.Diets effect on disease only when two or more statistical methods are in the same direction. |
|  | d) | Explain how missing data were addressed | NA |  |
|  | e) | If applicable, indicate how multiple testing was addressed |  | P values < 0.05 were considered nominally significant |
| 7 | Assessment of assumptions | Describe any methods or prior knowledge used to assess the assumptions or justify their validity | 3 | These selected SNPs are located in different gene regions and do not exhibit significant linkage disequilibrium (r2<0.001). Supplementary Table 6 contains additional detailed information related to the instrumental variables used in our study. |
| 8 | Sensitivity analyses and additional analyses | Describe any sensitivity analyses or additional analyses performed (e.g. comparison of effect estimates from different approaches, independent replication, bias analytic techniques, validation of instruments, simulations) | 15 | - Several sensitivity analyses were conducted to obtain stable MR estimates. The IVW and MR-Egger were utilised to quantify the heterogeneity effect among genetic instruments  - Cochran’s Q test assessed heterogeneity in the IVW model. Cochran’s Q test P<0.05 indicates the presence of heterogeneity  - Finally, the leave-one-out method was utilised to address sensitivity analysis. |
| 9 | Software and pre-registration |  |  |  |
|  | a) | Name statistical software and package(s), including version and settings used |  | All analyses were performed in R software (version 4.2.1) using the TwoSampleMR package. |
|  | b) | State whether the study protocol and details were pre-registered (as well as when and where) |  | NA |
|  | RESULTS |  |  |  |
| 10 | Descriptive data |  |  |  |
|  | a) | Report the numbers of individuals at each stage of included studies and reasons for exclusion. Consider use of a flow diagram | 15 | The baseline characteristics of the 345,313 (FTC), 347,429 (MT) and 346,859 (PTC) eligible participants were shown in Table 1. |
|  | b) | Report summary statistics for phenotypic exposure(s), outcome(s), and other relevant variables (e.g. means, SDs, proportions) | 12 | - Figure 3 & 4;  - Supplementary Table7 & 8. |
|  | c) | If the data sources include meta-analyses of previous studies, provide the assessments of heterogeneity across these studies |  | NA |
|  | d) | For two-sample MR:  i.  Provide justification of the similarity of the genetic variant-exposure associations between the exposure and outcome samples  ii.  Provide information on the number of individuals who overlap between the exposure and outcome studies |  | Supplementary Table8 |
| 11 | Main results |  |  |  |
|  | a) | Report the associations between genetic variant and exposure, and between genetic variant and outcome, preferably on an interpretable scale | 15-16 | - Figure 3 & 4;  - Supplementary Table 8. |
|  | b) | Report MR estimates of the relationship between exposure and outcome, and the measures of uncertainty from the MR analysis, on an interpretable scale, such as odds ratio or relative risk per SD difference | 15-16 | The causal effect estimate for each SNP on thyroid cancer is visualized using scatter plots of the outcome (Thyroid cancer) relationships and the exposure (diet factors) associations in Figure 3. The pooled results indicated that above the diet factors on the risk of thyroid cancer with low heterogeneity (Supplementary Table 8). Above all the findings are robust in the leave-one-out sensitivity analysis (Supplementary Figure 2). |
|  | c) | If relevant, consider translating estimates of relative risk into absolute risk for a meaningful time period | NA |  |
|  | d) | Consider plots to visualize results (e.g. forest plot, scatterplot of associations between genetic variants and outcome versus between genetic variants and exposure) | 15-16 | Figure 3A and 3B illustrates the beta values for various food intakes or nutrients in three type of thyroid cancer MR analysis with IVW methods. A positive beta value indicates a significant association between the exposure and outcome, with higher values representing a greater effect size. Figure 3C shows the estimated causal effects of different foods/nutrients on thyroid cancer, as well as a forest plot of the estimated values for each outcome using different MR methods (See detail result in Supplementary Table 7 and 8). |
| 12 | Assessment of assumptions |  | 15 | The pooled results indicated that above the diet factors on the risk of thyroid cancer with low heterogeneity (Supplementary Table 8). |
|  | a) | Report the assessment of the validity of the assumptions |  |  |
|  | b) | Report any additional statistics (e.g., assessments of heterogeneity across genetic variants, such as I2, Q statistic or E-value) |  |  |
| 13 | Sensitivity analyses and additional analyses |  | 15 | Above all the findings are robust in the leave-one-out sensitivity analysis (Supplementary Figure 2). |
|  | a) | Report any sensitivity analyses to assess the robustness of the main results to violations of the assumptions |  |  |
|  | b) | Report results from other sensitivity analyses or additional analyses |  |  |
|  | c) | Report any assessment of direction of causal relationship (e.g., bidirectional MR) |  |  |
|  | d) | When relevant, report and compare with estimates from non-MR analyses |  |  |
|  | e) | Consider additional plots to visualize results (e.g., leave-one-out analyses) |  |  |
|  | DISCUSSION |  |  |  |
| 14 | Key results | Summarize key results with reference to study objectives | 16 | In the MR analysis, we observed that Satsuma, Mushroom, Oily Fish,might be protective factors for thyroid cancer.However, cucumber, cooked vegetables, ham and cereal bar intake might be risk factors for thyroid cancer. |
| 15 | Limitations | Discuss limitations of the study, taking into account the validity of the IV assumptions, other sources of potential bias, and imprecision. Discuss both direction and magnitude of any potential bias and any efforts to address them | 17 | However, there are some limitations associated with MR as well. Although we selected the largest sample size and the most recent GWAS datasets available for our MR analysis, the sample size and number of events in our study were relatively small compared to those in population-based studies. Due to the generally low heritability of dietary exposure, which may limit statistical efficacy, future studies need to combine high-precision metabolomics or proteomics data to improve the explanatory power of instrumental variables. This study mainly explores the long-term average effect of dietary exposure. In the future, potential threshold effects can be explored by stratified MR analysis (e.g., stratified by intake) or nonlinear MR methods (e.g., quadratic model), which require a larger sample size to support. Additionally, our analysis was performed on populations with specific ancestries, which may introduce ascertainment bias. Consequently, these findings may not accurately reflect the broader population. Furthermore, the lack of demographic and detailed clinical information in the GWAS database hindered our ability to conduct subgroup analysis, limiting the depth of our insights. |
| 16 | Interpretation |  | 17 | Our MR results indicated that oily fish consumption may decrease the risk of developing thyroid cancer. Prior research has shown that moderate fish consumption does not significantly elevate the risk of thyroid cancer, and may even have a beneficial effect in areas with iodine deficiency , which is relatively consistent with our findings. In the study of thyroid cancer in women, Mack et al. found that shellfish and saltwater fish reduced risk independent of the risk of fish consumption, but our meta-study found no significant relationship between high shellfish and saltwater fish consumption and thyroid cancer risk [36]. One possible explanation is that iodine deficiency or excess may exist in the study area. In areas of severe iodine deficiency, a high intake of fish had a protective effect, whereas in areas of adequate iodine intake there was no effect . |
|  | a) | Meaning: Give a cautious overall interpretation of results in the context of their limitations and in comparison with other studies |  |  |
|  | b) | Mechanism: Discuss underlying biological mechanisms that could drive a potential causal relationship between the investigated exposure and the outcome, and whether the gene-environment equivalence assumption is reasonable. Use causal language carefully, clarifying that IV estimates may provide causal effects only under certain assumptions |  |  |
|  | c) | Clinical relevance: Discuss whether the results have clinical or public policy relevance, and to what extent they inform effect sizes of possible interventions |  |  |
| 17 | Generalizability | Discuss the generalizability of the study results (a) to other populations, (b) across other exposure periods/timings, and (c) across other levels of exposure | 1-2 | We also conducted an MR analysis to investigate the relationship between specific foods and thyroid cancer. Both meta-analysis and MR analysis found that fish was associated with a reduced risk of thyroid cancer. |
|  | OTHER INFORMATION |  |  |  |
| 18 | Funding | Describe sources of funding and the role of funders in the present study and, if applicable, sources of funding for the databases and original study or studies on which the present study is based | 18 | This research was supported by the Department of Science and Technology of Sichuan Province (No. 2024ZYD0193), Joint foundation of Chengdu Medical College and Jianyang Center for Disease Control and Prevention (No. 2022LHJY01), The project of Chengdu Pidu District People’s Hospital (No. 23LHPDZYB22) and the Open Fund of Sichuan Provincial Key Laboratory of Philosophy and Social Sciences for Intelligent Medical Care and Elderly Health Management (No.ZHYYZKYB2401). |
| 19 | Data and data sharing | Provide the data used to perform all analyses or report where and how the data can be accessed and reference these sources in the article. Provide the statistical code needed to reproduce the results in the article, or report whether the code is publicly accessible and if so, where | 18 | All data was provided in supplementary materials |
| 20 | Conflicts of Interest | All authors should declare all potential conflicts of interest | 18 | The authors declare that they have no competing interests. |

This checklist is copyrighted by the Equator Network under the Creative Commons Attribution 3.0 Unported (CC BY 3.0) license.
