# Supplementary material for: Dissecting the causal association of diet with thyroid cancer: a systematic review with meta-analysis and mendelian randomization analysis
Source: Front Nutr. 2025 Sep 17;12:1664129. doi: 10.3389/fnut.2025.1664129 (PMC12486595; doi:10.3389/fnut.2025.1664129)

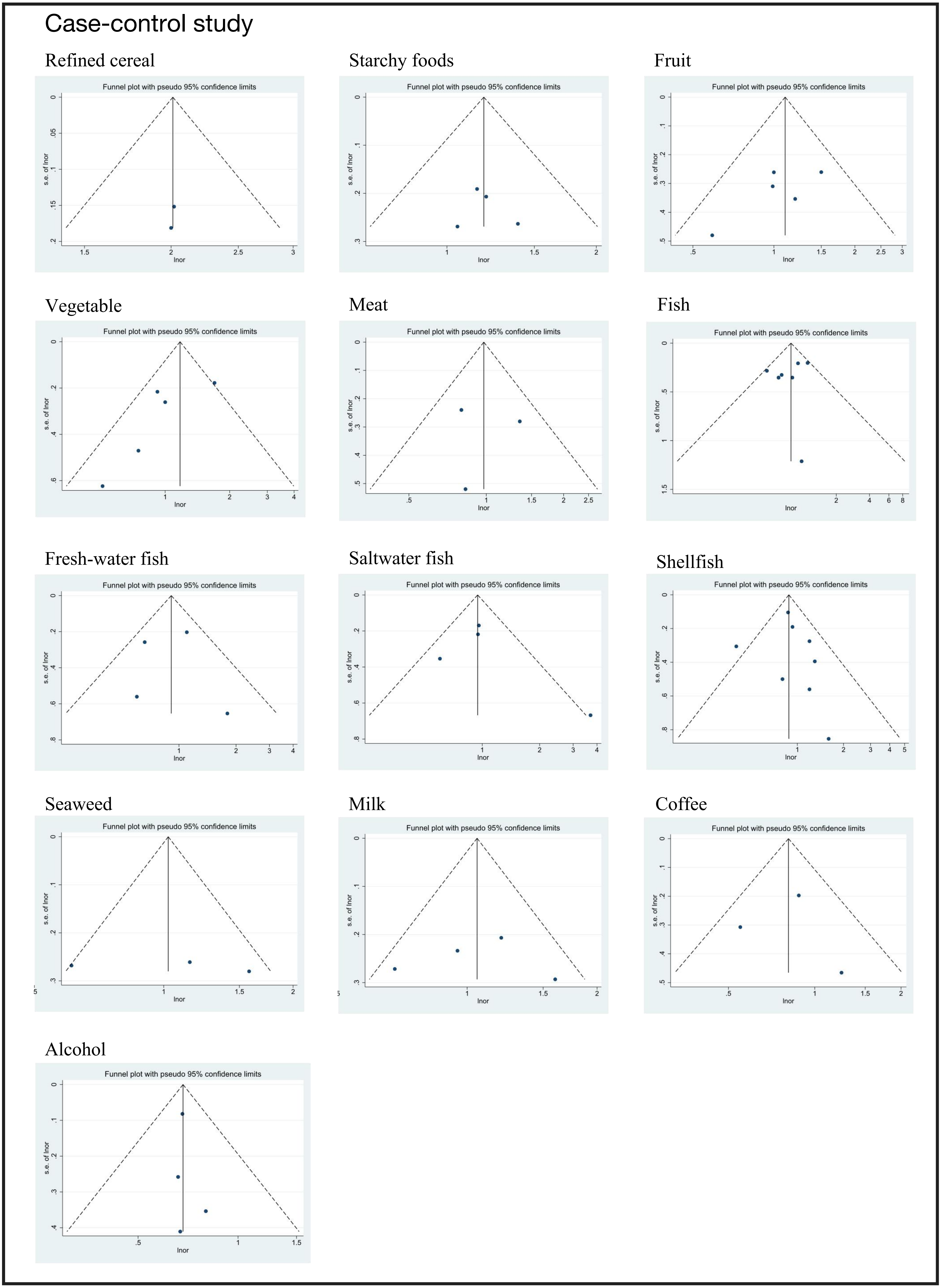
Case-control study
Refined cereal
Starchy foods
Fruit
Vegetable
Meat
Fish
Fresh-water fish
Saltwater fish
Shellfish
Seaweed
Milk
Coffee
Alcohol
Funnel plot with pseudo 95% confidence limits
s.e. of lnor
lnor

Cohort study

Fruit

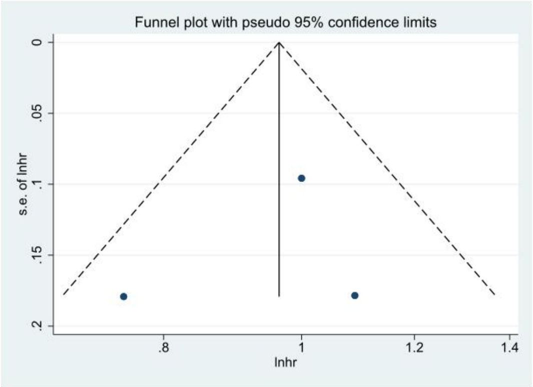

Vegetable

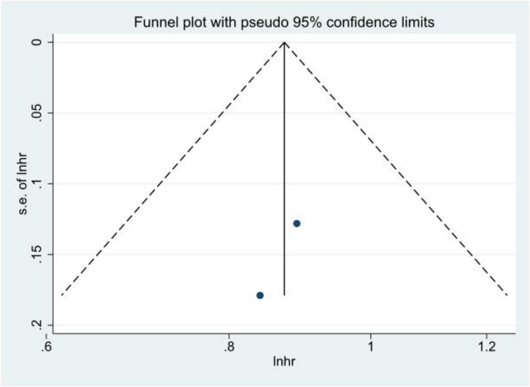

Meat

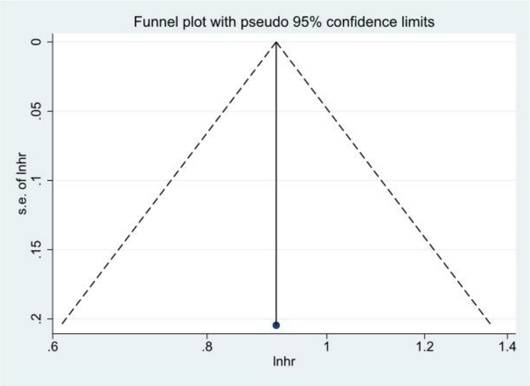

Seaweed

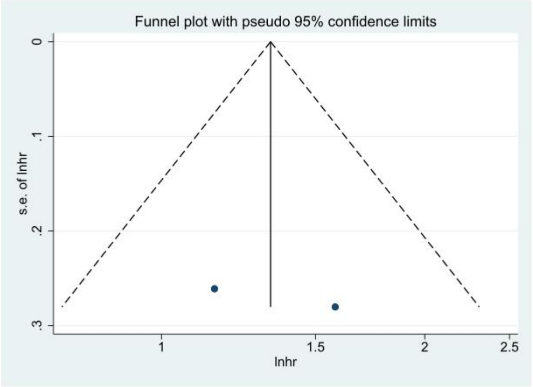

Tea

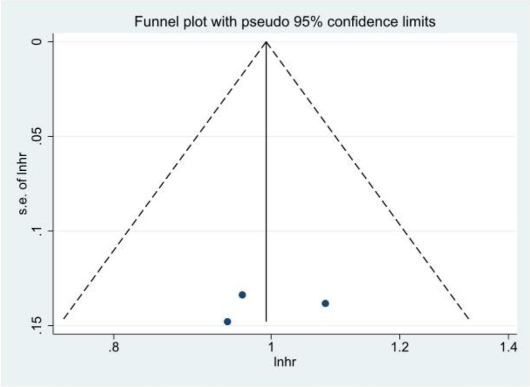

Coffee

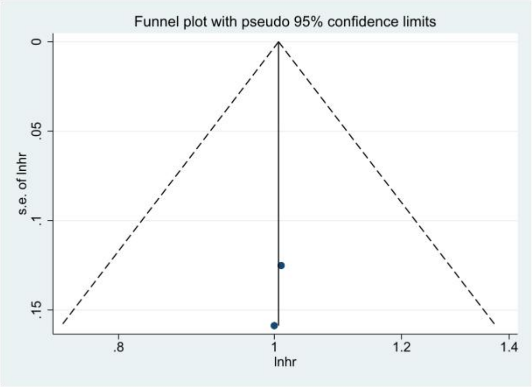

Alcohol

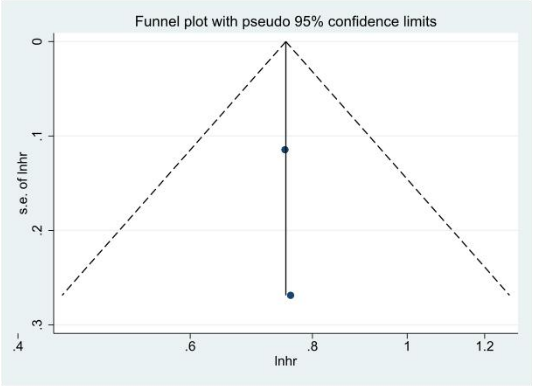

Nitrate

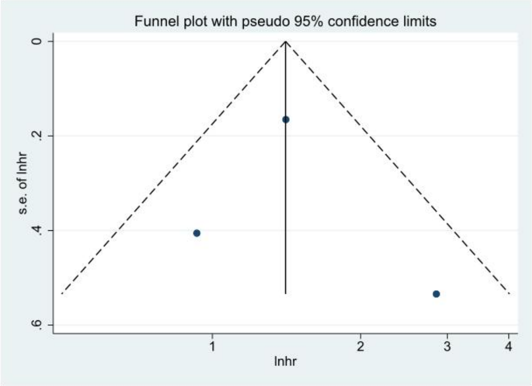

Supplement: Supplementary Figure 1 — Funnel plot detailing publication bias in the case-control and cohort studies reporting. [file Image_1.pdf]
